# Case Report: *ISPD* Gene Mutation Leads to Dystroglycanopathies: Genotypic Phenotype Analysis and Treatment Exploration

**DOI:** 10.3389/fped.2021.710553

**Published:** 2021-08-18

**Authors:** Haiyan Yang, Fang Cai, Hongmei Liao, Siyi Gan, Ting Xiao, Liwen Wu

**Affiliations:** ^1^Department of Neurology, Hunan Children's Hospital, Changsha, China; ^2^Department of Neurology, Chenzhou No. 1 People's Hospital, Chenzhou, China; ^3^Department of Pediatrics, Xiangya Hospital, Central South University, Changsha, China

**Keywords:** *ISPD* gene, dystroglycanopathies, limb-girdle muscular dystrophies, Duchenne muscular dystrophy, pediatric

## Abstract

*ISPD* gene mutation-related diseases have high clinical and genetic heterogeneity, and no studies have yet reported any effective treatments. We describe six patients with dystroglycanopathies caused by *ISPD* gene mutations and analyze their genotypes and phenotypes to explore possible effective treatments. Our results confirm that the phenotype of limb-girdle muscular dystrophies can be easily misdiagnosed as Duchenne muscular dystrophy and that exon deletions of *ISPD* gene are relatively common. Moreover, low-dose prednisone therapy can improve patients' exercise ability and prolong survival and may be a promising new avenue for *ISPD* therapy.

## Background

*ISPD* (MIM 614631, also named CDP-l-ribitol pyrophosphorylase A, *CRPPA*) gene, which maps to chromosome 7p21, has been associated with the loss of α-dystroglycan (α-DG) glycosylation (1–3). Defects in the post-translational modification of α-DG have been implicated in clinically distinct dystroglycanopathies that present as congenital muscular dystrophies with multisystem involvement, Walker–Warburg syndrome (MIM 614643), limb-girdle muscular dystrophies (LGMDs) (MIM 616052), or a spectrum of intermediate phenotypes (4). *ISPD* gene mutation-related diseases have high clinical and genetic heterogeneity (5). At present, there has been a lack of effective treatment for *ISPD* gene mutation-related diseases, and no studies have yet reported a possible effective treatment. Here, we report six patients with dystroglycanopathies caused by *ISPD* gene mutations and analyze their genotypes and phenotypes to explore possible effective treatments.

## Case Presentation

**Figure 1:**
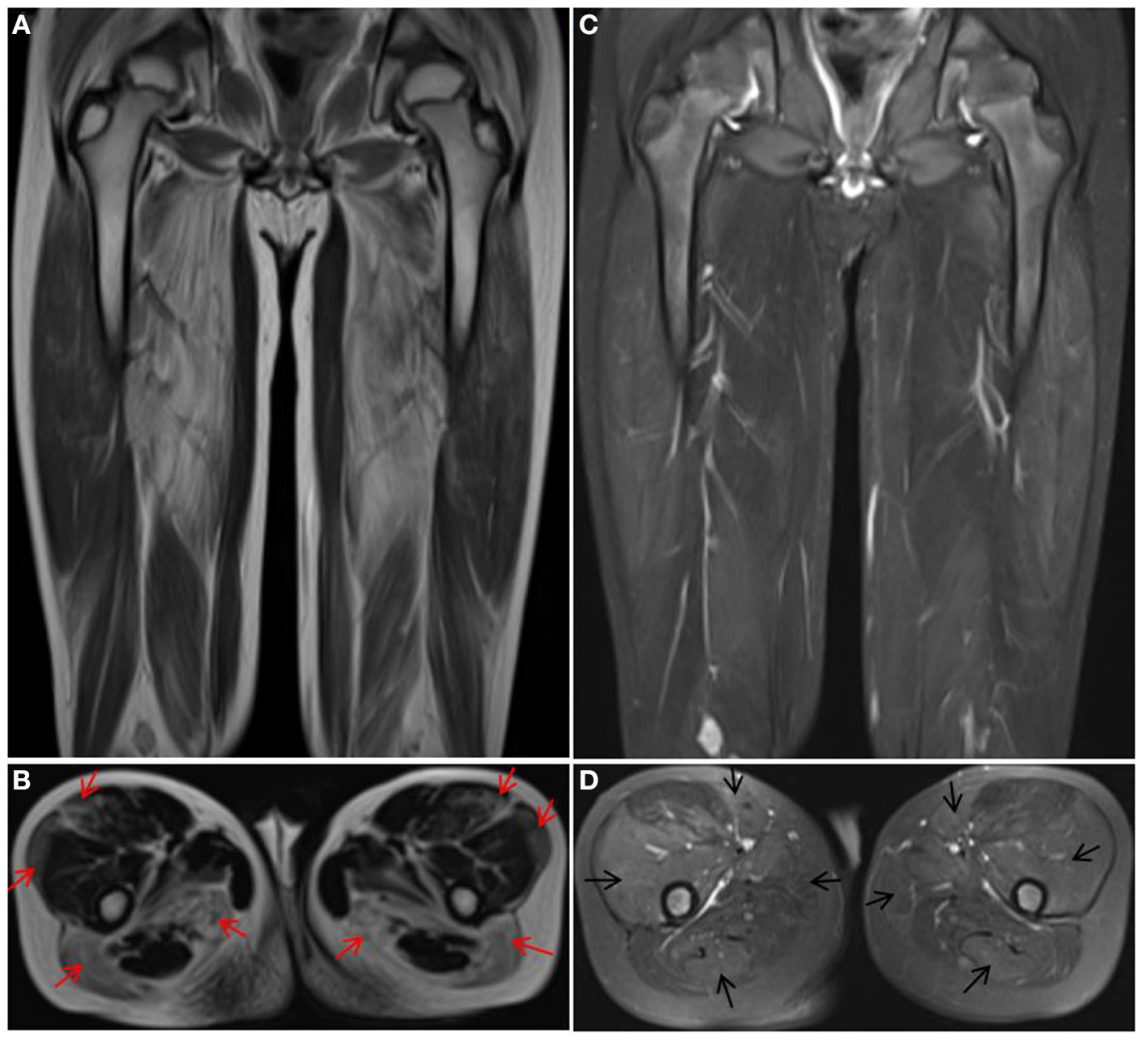
Muscle MRI of case 1. T1 **(A,B)** showed obvious fat infiltration in thigh muscles, most notably in the vastus lateralis, rectus femoris, biceps femoris, and semimembranosus muscles (red arrow), but the fat infiltration in the vastus intermedius, sartorius, gracilis, and semitendinosus muscles was mild. T2 **(C,D)** showed obvious inflammation edema in the muscles with mild fat infiltration (black arrow).

Two siblings (case 1 and case 2) were reported in this family. The younger brother (case 1) was a 9-year-old male child who presented with a history of progressive weakness of the lower limbs since age 1.5 years. The results of laboratory testing revealed elevated creatine kinase (CK) (13,043.5 U/L) and elevated lactic acid (2.30 mmol/L) levels. Muscle biopsy showed myodystrophy changes. Muscle MRI showed different degrees of fatty infiltration with partial inflammation edema in each muscle group of the thigh (Figures 1A–D). The initial clinical diagnosis was considered Duchenne muscular dystrophy (DMD). The child has received prednisone (0.75 mg/kg/day) treatment since the age of 8, and the symptoms of muscle weakness have improved, along with a longer walking duration and less wrestling. After genetic diagnosis, the younger brother gradually stopped prednisone treatment plan. However, after prednisone reduction, the patient's motor function decreased, and the wrestling increased. The symptoms improved again when the child resumed prednisone therapy. The older sister (case 2) presented with a history of progressive weakness after birth and never gained the ability to walk independently. At the age of 3, a muscle biopsy showed myodystrophy changes; she did not receive special treatment and died at the age of 16.

Case 3 was a 16-year-old male patient who presented with a history of progressive weakness of the lower limbs from childhood. The results of laboratory testing revealed elevated CK levels (7,431.5 U/L), and muscle biopsy showed myodystrophy changes. The initial clinical diagnosis was considered DMD. The patient has received prednisone treatment (20 mg, gradually increased to 35 mg, Qd, 10-day regimen followed by a pause for 20 days) since the age of 12, and the symptoms of muscle weakness have improved. The child has no obvious Gowers' sign and can jump 2–3 cm off the ground with both feet but can barely jump off the ground with one foot.

Case 4 was a 10-year-old male child who presented with a history of progressive weakness of the lower limbs since age 1.3 years and the loss of ambulation at age 10. The results of laboratory testing revealed elevated CK (6,329.6 U/L). Muscle biopsy showed myodystrophy changes. The child was not treated with any medication.

Two sisters were also identified in this family. The proband (case 5) was 5 years old, was female, and had normal development, but she had slight weakness when climbing and running. Her CK level was between 2,400 and 5,600 U/L. She had a 3-year-old sister (case 6) with the same motor development features who suffered from retinoblastoma, and her CK was between 1,000 and 8,800 U/L. Their mother was 32 years old, presenting with intolerance of movement and an elevated CK level of 600–4,500 U/L. Muscle biopsy of the proband revealed atrophy in parts of the muscle fibers, mild hyperplasia of connective tissue, negative dystrophin-N staining in most muscle fibers, and decreased expression of dystrophin-C and dystrophin-R. Except for the younger sister of the proband, who underwent ophthalmectomy, the proband was not treated with any medication.

## Methods

Written informed consent was obtained from the patients and their parents. This study was approved by the Medical Ethics Committee of Hunan Children's Hospital.

Genetic testing included the use of the multiplex ligation-dependent probe amplification (MLPA) method to screen for *DMD* gene duplication and deletion and next-generation sequencing methods to screen for other genes that might cause elevated CK and muscle weakness. All experiments and analyses were performed according to our previous research methods (6).

Sequence variants were annotated using population and literature databases, including 1,000 Genomes, dbSNP, GnomAD, Clinvar, HGMD, and OMIM. Variant interpretation was performed according to the American College of Medical Genetics and Genomics (ACMG) guidelines (7).

## Results

**Figure 2:**
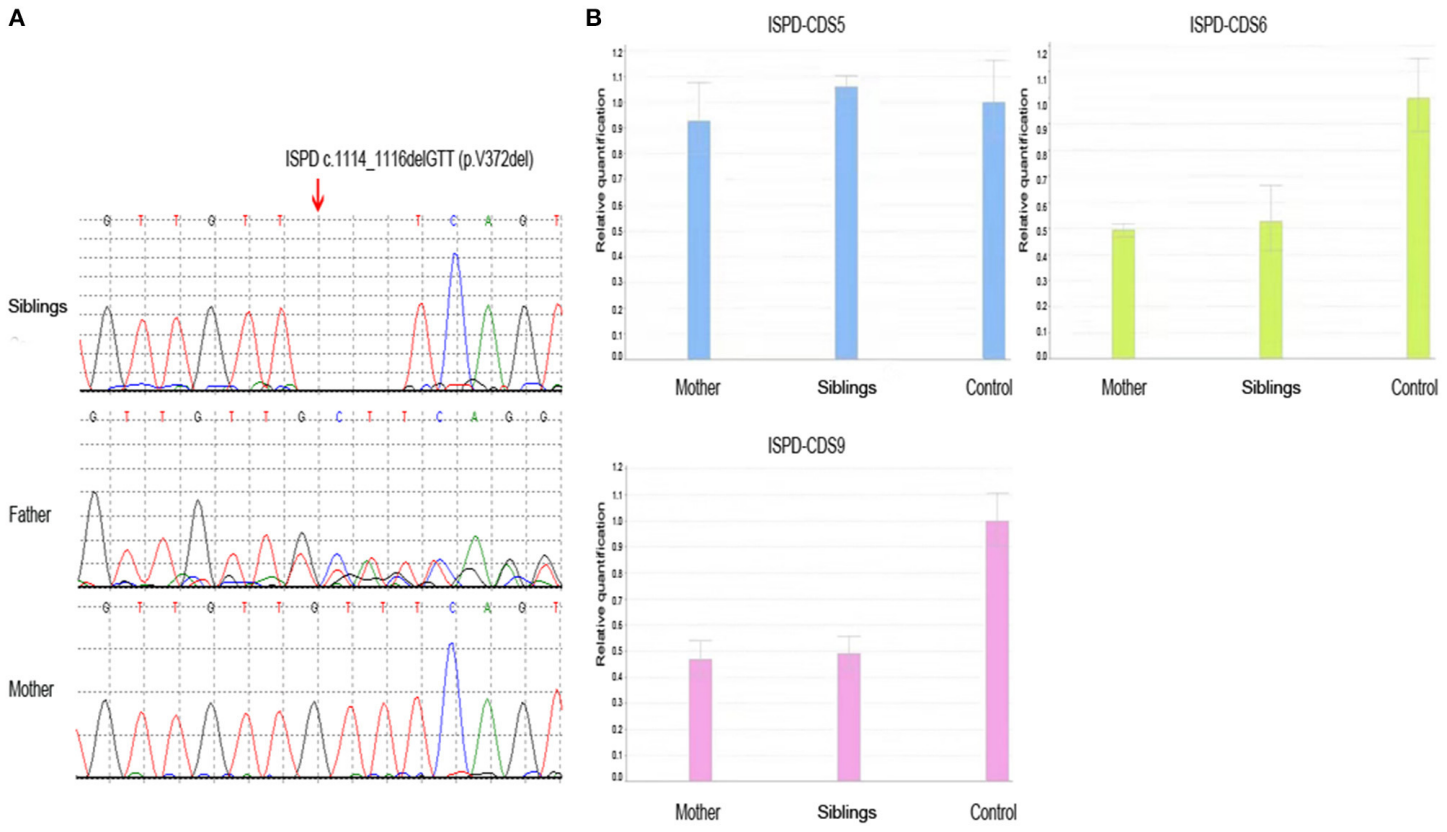
The result of whole-exome sequencing of the siblings. **(A)** The siblings had *ISPD* c.1114_1116delGTT (p.V372del) mutation, which was inherited from the father, while the mother did not carry the mutation. **(B)** The siblings had *ISPD* exon 6–9 deletion, which was inherited from the mother.

The detection for *DMD* gene from peripheral blood of the siblings (case 1 and case 2) was negative, including duplication and deletions, point mutations, and intron region mutations. Moreover, the possible RNA mutations of *DMD* gene were negative by detecting the muscle tissue of the siblings. These results were puzzling. Therefore, in September 2020, we reanalyzed the previous second-generation sequencing data and found that the siblings (case 1 and case 2) had *ISPD* c.1114_1116delGTT (p.V372del) mutation and exon 6–9 deletion (Figures 2A,B). The siblings (case 1 and case 2) were eventually diagnosed with LGMDs. The *DMD* gene duplication deletion, point mutation, and intron mutation test results of case 3 were all negative. Further next-generation sequencing revealed *ISPD* c.1114_1116delGTT (p.V372del) mutation, and the patient was eventually diagnosed as LGMDs. Case 4 had *ISPD* c.1114_1116delGTT (p.V372del) and *POMT1* c.979G>A (p.V327I) mutations. The sisters (case 5 and case 6) had *ISPD* c.984G>T (p.Q328H) and c.550C>T (p.R184X) compound heterozygous mutations and *DMD* c.130dupC mutation. Further details can be found in Table 1.

**Table 1:** Genotype and phenotype analysis of the 6 patients with *ISPD* mutation.

**Patient**	**Sex**	**Age**	**Muscle MRI**	**Muscle biopsy**	**Mutation**	**Inheritance**	**ACMG classification**	**Clinical feature**	**Effective treatment**	**Outcome**
1	M	9 y	Fatty infiltration with partial inflammation edema in the thigh muscles	Myodystrophy	*ISPD*c.1114_1116delGTT (p.V372del) Exons 6-9 deletion	Paternal Maternal	P LP	Limb-girdle muscular dystrophies	Prednisone	Survive
2	F	16 y	N/A	Myodystrophy	*ISPD* c.1114_1116delGTT (p.V372del) Exons 6-9 deletion	Paternal Maternal	P LP	Limb-girdle muscular dystrophies	None	Died
3	M	16 y	N/A	Myodystrophy	*ISPD* c.1114_1116delGTT (p.V372del)	Paternal	P	Limb-girdle muscular dystrophies	Prednisone	Survive
4	M	10 y	N/A	Myodystrophy	*ISPD* c.1114_1116delGTT (p.V372del) *POMT1* c.979G>A (p.V327I)	Paternal Parental	P Benign	Limb-girdle muscular dystrophies	None	Survive
5	F	5 y	N/A	Myodystrophy	*ISPD* c.984G>T(p.Q328H) c.550C>T(p.R184X) *DMD* c.130dupC	Paternal Maternal Maternal	VUS LP Benign	Limb-girdle muscular dystrophies	None	Survive
6	F	3 y	N/A	N/A	*ISPD* c.984G>T(p.Q328H)	Paternal	VUS	Limb-girdle muscular dystrophies	None	Survive
					c.550C>T(p.R184X)	Maternal	LP			
					*DMD* c.130dupC	Maternal	Benign			

*ACMG, American College of Medical Genetics; F, Female; LP, likely pathogenic; MRI, Magnetic resonance imaging; M, Male; N/A, Not available; P, pathogenic; VUS, variant of unknown significance; y, year*.

## Discussion

Relevant case reports from PubMed database since its establishment until December 2020 were searched with ISPD gene as the key word, and the variants and clinical phenotypes of ISPD gene were summarized. Currently, a total of 58 patients with *ISPD* gene mutations have been reported in nine literatures (2–5, 8–12) (Table 2). The reported clinical phenotypes include intrauterine growth restriction, gastroschisis, Walker–Warburg syndrome, LGMDs, cobblestone lissencephaly, and congenital muscular dystrophy with developmental delay. For LGMDs due to *ISPD* mutations, onset occurs in late childhood, adolescence, or adulthood (8). The main clinical manifestations of LGMDs include proximal muscle involvement of the upper and lower extremities, elevated CK, gastrocnemius hypertrophy, and Gowers' sign (13). Muscle biopsy and muscle MRI revealed changes indicative of myodystrophy, which is difficult to distinguish from DMD; and thus, these cases can be easily misdiagnosed (14).

**Table 2:** Genotype and phenotype analysis of the *ISPD* gene reported in literature.

**Mutation**	**Case (*n*)**	**Inheritance (*n*)**	**Sex (*n*)**	**Age (m)**	**Brain MRI (*n*)**	**Clinical feature**	**ACMG classification**	**Outcome**
c.367G>A (p.G123A)	2	Parental (2)	F (1)	4.3 y	*N* (2)	Limb-girdle muscular dystrophies	LP	Survive (2)
			M (1)					
7p21.2p21.1 microdeletion	3	Maternal (3)	Fetus (3)	N/A	*A* (2)	Intrauterine growth restriction; Gastroschisis	P	Survive (1)
					*N*(1)			Died (2)
Exons 9 and 10 deletion	1	Paternal (1)	M(1)	13 months	*A* (1)	Walker-Warburg syndrome	P	Died (1)
c.614G>A (p.A205H)		Unknown (1)					P	
c.161G>C (p.G54A)	2	Maternal (2)	F (1)	50 y	*A* (2)	Limb-girdle muscular dystrophies	P	Survive (2)
			M (1)					
c.1114_1116delGTT (p.V372del)	6	Parental (3)	F (5)	32 y	*N* (4)	Limb-girdle muscular dystrophies	P	Survive (6)
		N/A (3)	N/A (1)		N/A (2)			
Exons 9 and 10 deletion	1	Parental (1)	N/A	N/A	N/A	Walker-Warburg syndrome	P	N/A
Exons 6–8 deletion	1	Parental (1)	N/A	N/A	N/A	Walker-Warburg syndrome	P	N/A
c.647C>A (p.A216A)	1	Parental (1)	N/A	N/A	N/A	Walker-Warburg syndrome	P	N/A
c.832A>T (p.Lys278*)	1	Parental (1)	N/A	N/A	N/A	Walker-Warburg syndrome	P	N/A
c.1186G>T (p.Glu396* )	1	Parental (1)	N/A	N/A	N/A	Walker-Warburg syndrome	P	N/A
c.53dupT (p.Ser19fs)	1	Paternal (1)	N/A	N/A	N/A	Walker-Warburg syndrome	P	N/A
c.377G>A (p.A126H)		Maternal (1)					P	
c.364G>C (p.A122P)	1	Maternal (1)	N/A	N/A	N/A	Walker-Warburg syndrome	P	N/A
c.802C>T (p.Arg268*)		Paternal (1)					P	
c.638T>G (p.M213A)	3	N/A	Fetus (3)	N/A	A (3)	Cobblestone-Lissencephaly	LP	N/A
exons 3 to 6 deletion		N/A					N/A	
c.466G>A (p.A156A)	2	N/A	Fetus (2)	N/A	A (2)	Cobblestone-Lissencephaly	LP	N/A
c.713C>T (p.T238I)	1	N/A	Fetus (1)	N/A	A (1)	Cobblestone-Lissencephaly	LP	N/A
c.256A>T (p.Arg86*)		N/A					N/A	
c.257+2T>G (*)	1	*De novo*	Fetus (1)	N/A	A (1)	Cobblestone-Lissencephaly	N/A	N/A
c.773C>A (p.Ser258)		N/A					N/A	
c.676T>C (p.T226H)	1	N/A	Fetus (1)	N/A	A (1)	Cobblestone-Lissencephaly	LP	N/A
Exons 4 to 6 deletion		N/A					N/A	
c.643C>T (p.Gln215* )	2	N/A (2)	F (1)	7 y	N/A	Walker-Warburg syndrome	N/A	N/A
Exons 9 and 10 deletion			N/A					
c.789+2T>G	2	N/A	N/A	N/A	N/A	Walker-Warburg syndrome	N/A	N/A
Exon 4 deletion								
c.277_279del ATT (p.Ile93del)	1	N/A	N/A	N/A	N/A	Walker-Warburg syndrome	N/A	N/A
c.1354T>A (p.*452Arg)	1	N/A	N/A	N/A	N/A	Walker-Warburg syndrome	N/A	N/A
c.1120-1G>T	1	N/A	N/A	N/A	N/A	Walker-Warburg syndrome	N/A	N/A
Exon 9 deletion								
c.550C>T (p.Arg184*)	1	N/A	N/A	N/A	N/A	Walker-Warburg syndrome	N/A	N/A
c.5A>T (p.G2V)	1	Maternal (1)	N/A	N/A	N/A	Walker-Warburg syndrome	P	N/A
c.505A>T (p.Lys169*)	1	Paternal (1)	N/A	N/A	N/A	Walker-Warburg syndrome	N/A	N/A
c.340C>G (p.H114A)	1	Maternal and Paternal (1)	N/A	N/A	N/A	Congenital muscular dystrophy	P	N/A
c.464A>G (p.H155A)	1	Paternal (1)	N/A	N/A	N/A	Congenital muscular dystrophy	P	N/A
c.712A>G (p.T238A)	1	Maternal (1)	N/A	N/A	N/A	Congenital muscular dystrophy	p	N/A
c.659A>T (p.A220V)	1	Maternal (1)	N/A	N/A	N/A	Congenital muscular dystrophy	P	N/A
c.1251G>A (p.Val374-Gln417del)	5	Paternal (1)	N/A	N/A	N/A	Congenital muscular dystrophy	N/A	N/A
c.990delC (p.Ile331Serfs*2)	1	Maternal (1)	N/A	N/A	N/A	Walker-Warburg syndrome	N/A	N/A
exons 6-9 deletion	1	Maternal (1)	N/A	N/A	N/A	Congenital muscular dystrophy with mental retardation	N/A	N/A
c.1026+1G>A	2	Parental (2)	N/A	N/A	N/A	Limb-girdle muscular dystrophies	N/A	N/A
c.1124A>G	1	Maternal (1)	N/A	N/A	N/A	Limb-girdle muscular dystrophies	N/A	N/A
c.1186G>T (p.Glu396*)	1	Parental (1)	N/A	N/A	N/A	Congenital muscular dystrophy with mental retardation	N/A	N/A
c.1183A>T (p.Arg395*)	2	N/A (2)	F (2)	14.5 y	N (2)	Limb-girdle muscular dystrophies	N/A	N/A
c.377G>A (p.A126H)	1	N/A	M (1)	6.5 y	A (1)	Limb-girdle muscular dystrophies	N/A	N/A
c.677A>G(p.T226C)	1	N/A	F (1)	10 y	N (1)	Limb-girdle muscular dystrophies with mental retardation	N/A	N/A

*A, Abnormal; ACMG, American College of Medical Genetics; F, Female; MRI, Magnetic resonance imaging; M, Male; m, medium; N, Normal; N/A, Not available; n, number; p, pathogenic; y, year*.

At present, 48 variants of *ISPD* have been reported, of which point mutations account for 70.8% (34/48) and exon deletions account for 29.2% (14/48) (2–5, 8–12) (Table 2). Collectively, the literature and our experience with these patients indicate that exon deletion of *ISPD* gene is common. These microdeletions can be clinically difficult to detect by conventional second-generation sequencing methods. Therefore, for patients with LGMDs clinically excluded from DMD, in addition to routine screening for *ISPD* point mutations, we must carefully consider the possibility of *ISPD* exon deletion, which should be investigated during data analysis.

Among our patients, *ISPD* mutations were found to coexist with gene mutations of another myopathy. Compared with the other two male patients in this cohort who had only *ISPD* gene mutations, the male patient who had *ISPD* and *POMT1* gene mutations developed muscle weakness more quickly and lost walking ability at an earlier age. In the sisters with *ISPD* and *DMD* gene mutations, the degree of CK elevation was higher than that of their mother without synergistic *ISPD* pathogenic genes, and the symptoms of exercise intolerance appeared at an earlier age than their mother's. Therefore, whether *ISPD* mutation is synergistic with *DMD* or *POMT1* gene mutation should be carefully considered. These conditions increase the difficulty and complexity of prenatal diagnosis, so clinicians must be vigilant.

In our study, two patients (case 1 and case 3) were clinically diagnosed with DMD before genetic diagnosis and were offered the currently recommended low-dose prednisone therapy for DMD (15), which was unexpectedly found to be effective in improving the patient's motor function, prolonging the walking time and reducing the frequency of wrestling. The motor function of the two patients was improved compared with that before and after treatment and that of untreated case 4. After the genetic diagnosis, we also attempted to reduce and discontinue the hormone treatment for case 1. After the hormone treatment was discontinued, the patient's muscle weakness returned, and the frequency of wrestling increased significantly. After the hormone treatment was resumed, the symptoms improved again. Muscle MRI in case 1 showed significant inflammation edema changes before muscle atrophy, which further suggested that prednisone therapy could alleviate symptoms after improving inflammation edema.

In conclusion, the genotypes and phenotypes of *ISPD* gene mutations represent a wide spectrum, and the phenotype of LGMDs is easily misdiagnosed as DMD. Exon deletions of *ISPD* gene are relatively common, so caution must be taken in the selection of gene testing methods and data analysis to avoid false-negative results. *ISPD* and other gene mutations coexist in some patients, so the possibility of synergistic pathogenesis needs to be confirmed by further large sample studies and functional verification. Our findings indicate that low-dose hormone therapy can improve patients' exercise ability and prolong survival and may be a promising new avenue for *ISPD* therapy.

## Data Availability Statement

The original contributions presented in the study are included in the article, further inquiries can be directed to the corresponding author/s.

## Ethics Statement

The studies involving human participants were reviewed and approved by the Medical Ethics Committee of Hunan Children's Hospital. Written informed consent to participate in this study was provided by the participants' legal guardian/next of kin. Written informed consent was obtained from the minor(s)' legal guardian/next of kin for the publication of any potentially identifiable images or data included in this article.

## Author Contributions

HY conducted the literature review and drafted the manuscript. FC, HL, SG, and TX made substantial contributions to the conception and interpretation of data. LW was responsible for revising the manuscript critically and has given final approval of the version to be published. All authors read and approved the manuscript.

## Conflict of Interest

The authors declare that the research was conducted in the absence of any commercial or financial relationships that could be construed as a potential conflict of interest.

## Publisher's Note

All claims expressed in this article are solely those of the authors and do not necessarily represent those of their affiliated organizations, or those of the publisher, the editors and the reviewers. Any product that may be evaluated in this article, or claim that may be made by its manufacturer, is not guaranteed or endorsed by the publisher.
